# Interoception and body image in breast cancer patients: a mindfulness-based stress reduction protocol

**DOI:** 10.3389/fpsyg.2024.1394355

**Published:** 2024-10-14

**Authors:** Valeria Sebri, Silvia Francesca Maria Pizzoli, Chiara Marzorati, Ketti Mazzocco, Gabriella Pravettoni

**Affiliations:** ^1^Applied Research Division for Cognitive and Psychological Science, European Institute of Oncology IEO, IRCCS, Milan, Italy; ^2^Department of Oncology and Hemato-Oncology, University of Milan, Milan, Italy; ^3^Department of Psychology, Università Cattolica del Sacro Cuore, Milan, Italy

**Keywords:** mindfulness-based stress reduction, breast cancer survivors, interoception, body image, psychological intervention

## Abstract

Breast cancer impairs physical and psychological well-being, even some years after treatments. Oncological treatments can strongly affect the body due to scars and breast(s) removal, for example, increasing symptoms of anxiety and depression. Psychological studies are effective in improving breast cancer survivors’ emotions and behaviors through several approaches to interventions. Over years, the Mindfulness-Based Stress Reduction (MBSR) has been evaluated as an effective intervention to promote well-being in breast cancer survivors. The present study protocol aims to evaluate the effectiveness of a MBSR intervention in regulating interoceptive sensations, as the ability to be aware of inner sensations. Second, it seeks to identify changes in interoceptive feelings, mood, and body perception following the intervention. These changes will be evaluated across three data collection times to assess differences about emotions and body perception over time, focusing on their relevance for breast cancer survivors’ well-being. Finally, the present study protocol aims to detect improvements in anxiety, depression, and body awareness, considering the potential positive impact of the MBSR approach on emotional well-being. Direction for future psychological intervention are given.

## Introduction

Most women with breast cancer have to deal with diagnosis and its related oncological treatments as a very difficult experience to accept, starting with the consequences on their Quality of Life (Schell et al., 2019). Oncological treatments (e.g., chemotherapy, radiotherapy, immunotherapy) can damage the body due to the presence of scars, hair loss, and breast(s) removal, for example (Fioretti et al., 2017). Therefore, patients generally report high levels of distress, anxiety, depression, physical symptoms of pain, fatigue and sleep–wake rhythm dysfunction, even several years after finishing treatments (Abrahams et al., 2018). Concerning emotional issues, it is mandatory to address the bodily self, defined as an expression of implicit knowledge about physical actions and movements (Sebri et al., 2021). Addressing bodily self is particularly relevant for breast cancer survivors, who concern changes in body image perception due to altered weight, possible hair loss, and the sudden onset of menopausal symptoms (Pintado and Andrade, 2017; Sebri et al., 2022). Additionally, body perception and body image are strictly tied to social and intimate relationships. Following the Discrepancy Theory by Higgins (1987), when there is a significant gap between one’s current and the desired body, it can lead to feelings of dissatisfaction and emotional distress. This discrepancy can severely impact women’s sense of femininity and sexuality (Male et al., 2016). Moreover, one’s own body after diagnosis and oncological treatments can be experienced as ill and damaged, even perceiving them as a source of danger for the fear of cancer recurrence. This can increase the risk of being labeled as “patients” rather than “persons,” reinforcing this type of self-definition and representation (McGannon et al., 2016). To improve levels of psycho-emotional well-being, the literature highlights the effectiveness of multimodal interventions aimed at improving both self-image perception and psycho-social aspects (Lengacher et al., 2009). Current studies demonstrated the efficacy of complementary and alternative interventions in groups of breast cancer survivors to improve positive social support (Durosini et al., 2021; Sebri et al., 2023; Sebri and Pravettoni, 2023). More specifically, numerous studies show the effectiveness of integrating standardized techniques and complementary medicine therapies, with a view to a multidisciplinary approach. Among them, one method effectively applied in the oncology field, and specifically in breast cancer patients (Carlson et al., 2004; Henderson et al., 2013), is the application of the Mindfulness-Based Stress Reduction (MBSR) protocol developed by Kabat-Zinn (1979).

Mindfulness is defined as paying attention to the unfolding of experience moment by moment, with intention and in a non-judgmental manner (Segal et al., 2014), cultivating a stable and non-reactive awareness even in stressful situations, such as that induced by a course of illness (Guu et al., 2023). Mindfulness allows one to pause ruminating thoughts, and access a greater awareness of one’s mental habits and related bodily sensations (Mehling et al., 2009; Pintado and Andrade, 2017). In this regard, the perception and awareness of what is happening within the body (e.g., concerning a general feeling of pleasantness/unpleasantness) is referred to as interoception (Barrett, 2017). Most Mindfulness-based protocols permit to cultivate interoceptive awareness, which plays a crucial role in the effectiveness of mindfulness-based interventions (Fischer et al., 2017), allowing the practitioners to shift attention from thinking about one’s bodily sensations to immediately feeling them (Farb et al., 2015; Mehling et al., 2017). Indeed, mindfulness can improve the relationship with interoceptive signals, encouraging more adaptive behaviors toward bodily sensations and reducing experiential avoidance (Weng et al., 2021). Accordingly, individuals who have practiced mindfulness report changes in their interoceptive awareness, having learned to redirect attention to their body, manage stress, regulate emotions and facilitate cognitive insight into their body/emotional state (de Jong et al., 2016; Fissler et al., 2016). Furthermore, enhanced interoception is also linked to improved mental health outcomes, making it a relevant and valuable capacity targeted in MBSR interventions for breast cancer patients.

Increased emotional awareness is indeed associated with a decrease in somatic symptoms (such as nausea, pain and fatigability) and distress, as well as a subsequent reduction in mortality in breast cancer patients (Czamanski-Cohen et al., 2019). Specifically, previous studies noted increased vitality, decreased physical pain, improvements in both mood (e.g., anxiety and depression) and social functioning (Reibel et al., 2001). The MBSR program also facilitates other ways of taking care of oneself (e.g., more rest and physical activity), paying attention without evaluation or judgment (Glynn et al., 2020). Accordingly, it provides greater benefits compared to usual care across all diagnoses, except for cognitive-behavioral therapy that is considered more efficacy in some specific cases (e.g., pain management) (Pardos-Gascón et al., 2021). Comparisons between mindfulness interventions are scarce, with MBSR being the most studied. In central sensitization syndromes, variables associated with pain tend to improve with treatment (Pardos-Gascón et al., 2021). Finally, regarding body perception, some studies report greater acceptance of oneself and one’s body after MBSR interventions (Luiggi-Hernandez et al., 2018; Penlington, 2019; Tate et al., 2018; Visser et al., 2015). Indeed, the effectiveness of MBSR programs about changes in body image perception is noted, highlighting how increased awareness can positively impact self-image. In this regard, a study by Pérez-Peña et al. (2022) highlighted the impact of mindfulness on proprioception within the body image perception. Proprioception is defined as the perception of body movement and position. It depends on both psychological (e.g., memory and learning) and physiological processes (e.g., mechanosensory neurons throughout the body known as proprioceptors) (Tuthill and Azim, 2018). Similar to interoception, proprioception is largely unconscious, although some proprioceptive information can be brought into conscious awareness (Pérez-Peña et al., 2022).

Even though the cancer experience causes quite a few issues related to the perception of self and one’s internal states, few programs specifically aimed at improving body image have been developed so far (Pintado and Andrade, 2017; Pidgeon and Appleby, 2014). To date, few studies have analyzed the effectiveness of MBSR programs in treating body image in breast cancer patients. In a recent randomized trial (Pintado and Andrade, 2017), patients who followed an MBSR protocol reported a decrease in negative thoughts and emotions related to self-image perception, an increase in positive thoughts, and increased body awareness. However, the scientific literature has so far not focused on measuring the effectiveness of MBSR protocols on interoceptive awareness in breast cancer patients. The overall aim of the present study is to evaluate the effectiveness of a MBSR intervention in regulating interoceptive feelings in women with breast cancer experience. This study will highlight possible changes in emotions and body perception in breast cancer survivors. Specifically, we aim to identify predictive aspects of the intervention on interoceptive feelings, mood, and body perception according to the three data collection times. In addition, we expect to be able to detect an improvement in mood (anxiety and depression) and body awareness (medium-term benefit).

## Methods

The present protocol was obtained from a licensing committee from a University that approved the study. Additionally, all participants signed the informed consent form. Lastly, this study follows the Declaration of Helsinki and method design will be carried out in accordance with relevant guidelines and regulations. Participants who belong to breast cancer associations will be contacted and invited to take part in this project by email. If they will accept, an informed consent before starting the first meeting will be sent.

The inclusion criteria for participation in the study are as follows:

1. Age between 30 and 50 to guarantee the sample homogeneity;
2. Patients with the first diagnosis of stage I, II, and III breast cancer;
3. Patients who have experience of previous surgery as an oncological intervention to remove cancer.
4. Patients who have received chemotherapy and completed treatment. Only chemotherapy can be accepted as the oncological treatments, not immunotherapy or radiotherapy. This criterion is chosen to ensure the homogeneity of the sample and to focus on patients who are not in an advanced stage of therapy but have completed their treatments.
5. Fluently understand and speak the Italian language.

Exclusion criteria are related to:

1. Metastatic disease, meaning the need to treat other types of cancer in addition to breast cancer;
2. Patients with a previous cancer diagnosis who have developed other types of cancer, different from breast cancer;
3. Patients who are undergoing psychological treatment, whether individually or in groups. It is relevant to ask them if they are currently participating in a psychological intervention, especially if it involves processing of cancer-related issues;
4. Presence of neurological deficits and/or psychiatric disorders that could impair rationale participation to the study;
5. Lack of knowledge of the Italian language, which could affect the full and accurate understanding of both the mindfulness practice and the questionnaires’ contents.

This study will last approximately 6 months and will use a quantitative research methodology, with a between-subjects design. Forty participants meeting the inclusion and exclusion criteria set out above will be enrolled and randomly distributed through Excel software (RAND function) into the following two groups:

*Experimental group*: participants who will be immediately included in the MBSR protocol;

*Waitlist control group*: participants who will be included in the MBSR protocol after 12 weeks from the start of the protocol.

The intervention proposed to the experimental group will be conducted by a psychologist expert in the MBSR protocol. The MBSR intervention will be structured as follows: it envisages eight weekly group meetings lasting about 2/2.5 h each, plus an intensive meeting (generally proposed between the sixth and seventh meetings or between the seventh and eighth meetings), which lasts about 5 h. Moreover, a follow-up meeting about a month after the conclusion of the course will be proposed. Participation in the course is preceded by an acquaintance and information meeting with the trainer who will be the course leader (40 min). The program will be experiential; participants do not passively receive guidance on how to manage stress but become active participants through systematic training in mindfulness practice. Specifically, based on the definition of mindfulness, the MBSR protocol invites people to cultivate awareness through meditation and teach how to enter into a different relationship with suffering, regardless of the specific causes, whether medical or psychological. It is possible to hypothesize that insights gained during meditative practice could serve as a basis for developing effective strategies for managing chronic pain conditions (Giommi, 2006).

The study will take place in a brightly lit and spacious room. This room will be specifically dedicated to accommodate group sessions, mindfulness exercises and group practices, with ample space for participants to either sit on the floor or use chairs, ensuring comfort and flexibility. All sessions will consistently be conducted in this same room, providing a stable and familiar environment for participants throughout the study.

On a practical level, the MBSR protocol will include (see Table 1 for a detailed schedule of meetings):

1. Moments of formal practice (sitting meditation with a focus on breath, body, sound, and mind content; body scan; mindful movement; walking meditation. These points will be discussed in detail below);
2. Free sharing of experiences and inquiry;
3. Formal practice to be performed daily at home with the support of audio tracks;
4. Informal practice (bringing awareness into everyday actions that we generally repeat daily according to automatic patterns);
5. Printed material to support formal and informal practice.
6. The formal and informal practice that the participant will be invited to perform daily takes approximately 40 min per day.

**Table 1:** Detailed description of the proposed meetings.

**Meeting**	**Activity**
**Session 1**	The first session will focus on presenting the activities to all participants and then introducing preliminary exercises to start the practice. Specifically: The facilitator will share preliminary directions and instructions with the group. A brief period of silence will follow A brief introduction to the definition of mindfulness will be provided to ensure participants understand its content Initial exercises will include a body scan followed by an inquiry, and participants will be guided to focus on their breath for 3-4 minutes The facilitator will assign homework related to the activities covered in the session The session will conclude with a final moment of silent reflection
**Session 2**	In the second session, participants will first be invited to practice the activities learned previously. Following this, additional mindfulness content will be introduced, as outlined below: After a brief introduction to the session's content and objectives, participants will engage in a body scan practice. They will then have the opportunity to discuss any questions about the body scan exercise performed at home and during the session, to enhance the effectiveness of the activity Next, the facilitator will guide participants in developing conscious awareness of their physical movements and sensations. Participants will be encouraged to ask questions, and then triads will be formed to discuss informal practices (e.g., brief exercises for individual practice at home) A group sitting meditation focused on breath awareness will be conducted for 15 minutes The facilitator will assign homework related to the activities covered in the session. In particular, participants will be invited to compile a diary with their "pleasant events" in the next days The session will conclude with a final moment of silent reflection
**Session 3**	In the third session, additional mindfulness content will be introduced and practiced in groups. Specifically: The session will begin with a brief introduction to its content and objectives. The psychologist will then lead a 20-minute seated meditation, focusing on body and breath. Afterward, participants will have the opportunity to discuss any questions about the formal practice they have completed at home. Participants will then engage in a 20-minute walking meditation. Next, a group discussion will be held to reflect on and share individual experiences of the previous practices. Triads will then take turns sharing from their "pleasant events" diary, compiled throughout the week. The psychologist will introduce the "triangle" map of mindfulness, a psychoeducational technique, to enhance understanding. The facilitator will assign homework related to the activities covered in the session The session will conclude with a final moment of silent reflection
**Session 4**	In the fourth session, participants will engage in additional mindfulness practices as follows: The session will start with a brief introduction to its content and objectives. The psychologists will then lead a 30-minute sitting meditation, focusing on body, breath, and sound. Participants will have dedicated time to discuss any questions about the formal practice they completed at home A new practice will be introduced, specifically standing and/or floor yoga Triads will take turns sharing from their "pleasant events" diaries, compiled over the past week A designated time will be allocated for discussing inquiries about unpleasant events, with a focus on identifying 'symptoms in the body' The psychologist will conduct a psychoeducational training on the stress response (first part) The facilitator will assign homework related to the activities covered in the session The session will conclude with a final moment of silent reflection
**Session 5**	In the fifth session, no new exercises will be introduced. Instead, participants will be encouraged to reinforce the valuable activities they have already learned. Additionally, the session will focus on discussing individual responses to stressful events and exploring appropriate strategies, as follows: The session will start with a brief introduction to its content and objectives. The psychologists will then lead a 40-minute sitting meditation, focusing on body, breath, and sound. Participants will have dedicated time to discuss any questions about the formal practice they completed at home Next, a group discussion will be held to reflect on and share individual experiences of the previous practices. Triads will then take turns sharing from their "pleasant events" diary, compiled throughout the week. In particular, participants will be invited to reflect on their reaction to stressful events, rescuing the information collected in the fourth session The psychologist will conduct a psychoeducational training on the stress response (second part) The facilitator will assign homework related to the activities covered in the session The session will conclude with a final moment of silent reflection
**Session 6**	The session will start with a brief introduction to its content and objectives. The psychologists will then lead a 40-minute sitting meditation, focusing on body, breath, and sound. Participants will have dedicated time to discuss any questions about the formal practice they completed at home Next, a group discussion will be held to reflect on and share individual experiences of the previous practices. Triads will then take turns sharing from their "pleasant events" diary, compiled throughout the week. In particular, participants will be invited to reflect on their communication with others, as a new focus of interest The facilitator will propose the first interpersonal awareness exercise, which have to conducted in pairs The facilitator will assign homework related to the activities covered in the session The session will conclude with a final moment of silent reflection
**Session 7**	This session will specifically focus on the second exercise related to interpersonal awareness, in addition to the usual practice of meditation and breath awareness. Participants will be invited to: The session will start with a brief introduction to its content and objectives. The psychologists will then lead a 40-minute sitting meditation, focusing on body, breath, and sound. Participants will have dedicated time to discuss any questions about the formal practice they completed at home Next, a group discussion will be held to reflect on and share individual experiences of the previous practices. Triads will then take turns sharing from their "pleasant events" diary, compiled throughout the week. In particular, participants will be invited to reflect on their communication with others, following the contents and reflections shared in the previous sessions The facilitator will propose the second interpersonal awareness exercise, which have to conducted in pairs The facilitator will assign homework related to the activities covered in the session The session will conclude with a final moment of silent reflection
**Session 8**	In the eighth and final session, participants will engage in new meditative exercises suitable for individual practice. Additionally, a final discussion about the program's content will be held. Specifically: The session will begin with a brief introduction to its content and objectives The psychologists will then lead a 40-minute body scan exercise Following this, the psychologists will conduct a session focusing on body, breath, sounds, thoughts, and open awareness. Participants will have time to discuss any questions about the formal practices completed at home After a brief summary of the course by the instructor, there will be a discussion on personal practice following the conclusion of the protocol, including inquiries about obstacles and motivations for continuing. The instructor will encourage individual self-reflection on the experience and significance of the course The session will conclude with a brief meditative writing exercise lasting approximately 10-15 minutes The session will conclude with a final moment of silent reflection

### Formal practice

MBSR intervention will be based of some formal practices, as follows:

- *Sitting meditation* consists of sitting while maintaining a composed, upright, yet not rigid posture, with head, neck and back aligned along a vertical axis, shoulders relaxed and hands in a comfortable position. This posture allows one to practice an attitude of dignity, patience, and calmness, bringing attention to the sensations emerging in the present moment without trying to fill time. For these reasons, it is necessary to dedicate time and space to the practice. The sitting meditation practice proposed in the first meetings of the MBSR pathway consists of directing attention with intention to the breath, remaining present to the sensations that accompany each inhale and exhale, without changing it. During meditation, the mind can become distracted and drift away from the object of attention; participants are asked To pay attention firmly and gently to one’s own breath, training the mind to be less reactive and more stable. This approach allows us to embrace each moment, cultivating concentrated calm, managing the mind’s resistance, and developing acceptance, patience, absence of judgment, and inner strength. It represents a training that teaches to deliberately let go of one’s thoughts, disidentifying from their content. In the course of the MBSR program, the practice of sitting meditation gradually broadens the field of attention to other objects besides the breath: sensations in various parts of the body and the body as a whole, sounds, and the process of thoughts. Recognizing thoughts as such allows us, as Kabat-Zinn (1979, 1997, 2016), to free ourselves from the distorted reality they can create, enabling us to manage our lives with greater fluidity. Similarly, dedicating time to practice daily allows us to strengthen our awareness, recognizing and accepting ourselves as we are and not as we would like to be;
- The *body scan* is a meditative practice that allows us to reconnect with the body, exploring it through a meticulous examination aimed at developing concentration and flexibility of attention. It is performed by lying on one’s back and systematically directing attention to various parts of the body. During the practice, participants are invited to inhabit each area of the body with full awareness, the sensations they are experiencing as well as their absence, attuning oneself as best as one can with the experience of the part of the body, which is the object of attention. The goal is to feel each part of the body and cultivate a non-judgmental, a moment-to-moment condition of awareness. Kabat-Zinn (1997) stated that the practice of body scan allows many practitioners to perceive their bodies positively; in particular, directing attention, kindness, and acceptance toward the own body becomes a valuable experience for what he describes as self-healing.
- The practice related to *conscious movement through yoga* consists of slowly performing and maintaining for a time a series of stretches of muscles and joints, while also continuously bringing attention to the breath and the current sensations. Kabat-Zinn (1997) describes this formal practice as an effective method to know ourselves more deeply, whatever our health conditions. During the conscious practice of yoga, there are no efforts or goals to achieve. On the contrary, this practice allows to grasp and get closer to physical and psychological limits, without having to cross or overcome them, but practicing patience with ourselves and observing the habitual way we deal with them;
- The *walking meditation* practice, in which attention is directed to the experience of walking in the moment and slow motion. More precisely, one chooses the place where to carry out the practice (indoors or outdoors); one defines the straight path to be followed (beginning and end); one stands still at the starting point of the path, for a few seconds, with feet parallel and apart, knees slightly flexed, arms stretched out along the sides or hands intertwined at belly level, the gaze unfocused in front of one. The invitation is to bring attention to the soles of the feet, to the physical sensations of contact with the floor, to the distribution of body weight during the steps taken, to the movement of the legs and the entire walking body, aiming at being present with each step, aimlessly, letting go of all thoughts. Being a moving practice, participants experience how the body keeps us anchored in the here-and-now through the grounding of the feet, while our mind tends to shift between the past and future. Participants are invited to practice walking meditation daily.

### Intensive meeting

The intensive meeting will include several formal meditation practices conducted and supervised continuously by the trainer. It is usually conducted between the sixth and seventh meetings or between the seventh and eighth meetings of the study. At this time, participants have been exposed to all the formal mindfulness practices; this way, they will be able to experience them in a similar way as during silent meditation retreat days. The intensive meeting will also become a time to break the rhythm of the usual routine by paying attention to the present moment. In other words, it will be an opportunity to empower the practice of mindfulness as an invitation to turn toward one’s suffering and observe more clearly the possible mental patterns that can exacerbate it. Specifically, a sequence of practices adapted to the group of participants is usually proposed, such as: sitting meditation practices focusing on breath, body, sounds, thoughts, or non-selective meditation practices; slow conscious movements to allow space for listening to one’s body and present sensations; body scan; walking meditation. Furthermore, informal meditation practices such as mindful eating will also be offered. The trainer will invite each participant to observe his or her own experience of food during lunch (which is done more slowly than usual, devoting more time to curiously observing the sensations present during the stages of tasting, chewing, swallowing).

### Evaluation tools

A battery of standardized and self-administered questionnaires will be administered through Qualtrics platform at the beginning and end of the MBSR intervention. The questionnaires will be administered to both groups in three stages: pre-intervention (T0), after 8 weeks (T1) and after 12 weeks (T2) from the beginning of the study. After the reading of the information sheet and the signing of the informed consent, participants will be asked to answer some socio-demographic and disease status questions, and then they will be asked to answer standardized and validated questionnaires designed to investigate interoceptive sensations, metacognitive and dissociative skills, sleep quality, state anxiety and depression, general well-being, and body perception. The duration of the questionnaire administration will be approximately 30–40 min.

Specifically, the questionnaires administered will be:

#### The multidimensional assessment of interoceptive awareness (MAIA)

MAIA is a self-report questionnaire that assesses eight dimensions related to interoceptive aspects of body awareness (Mehling et al., 2012). The scale includes a total of 32 items on a 6-point Likert scale, ranging from 0 (never) to 5 (always) and covers eight “distinct but related” dimensions of interoception: awareness of bodily sensations (e.g.: “I can tell where I feel good in my body”), the tendency to ignore uncomfortable bodily sensations (e.g., “I distract myself when I feel uncomfortable or fearful sensations”), the ability to have emotional reactions as a result of negative sensations (e.g., “I worry if I feel pain or uncomfortable sensations”), the ability to regulate attention regarding multiple sensations (e.g., “I can focus sensations on my body, even when there are many distractions around me”), the ability to be aware of the relationship between bodily and affective states (e.g., “I can feel changes in my body when I am happy”), the ability to pay attention to bodily states to regulate psychological distress (e.g., “I can use my breath to help me stay calm and relaxed), the ability to feel one’s bodily sensations to make decisions (e.g., “I listen to my body to help me choose what to do”), and the experience of one’s body as safe and trustworthy (e.g., “I feel that my body is a safe place). Some subscales measure direct experience with the body, others are associated with the assessment of cognitive processes, such as self-regulation (Ferentzi et al., 2020). The total score is calculated between 0 and 160 (Valenzuela-Moguillansky and Reyes-Reyes, 2015) and the reliability for the eight subscales (Mehling et al., 2012) is between 0.66 and 0.87.

#### The Italian version of the metacognitions questionnaire (MCQ-30)

MCQ-30 is a questionnaire with 30 items distributed on a 5-point Likert scale (from “not at all agree” to “completely agree”) (Quattropani et al., 2014). It measures dysfunctional metacognition processes regarding vulnerability and maintenance of emotional disorders through five categories: (1) positive beliefs regarding worry, describing the positive evaluation of worry as being functional for planning and solving problems (6 items, e.g., “I need to worry in order to work better”); (2) negative beliefs regarding uncontrollable and dangerous aspects, assessing the negative interpretation of worry due to thoughts considered uncontrolled (6 items, e.g.: “When I start worrying, I cannot stop”); (3) cognitive confidence, in order to explore concerns related to the effectiveness of one’s memory (6 items, e.g., “I do not trust my memory”); (4) the need to control thoughts, which assesses the belief that some thoughts need to be controlled and suppressed (6 items, e.g., “I should keep my thoughts under control all the time”); (5) cognitive self-awareness, which assesses the tendency to constantly pay attention to one’s thought processes (6 items, e.g., “I am constantly aware of my thoughts”). MCQ-30 shows good psychometric properties with reference to satisfactory internal consistency, convergent validity, and good test–retest reliability (Laghi et al., 2020).

#### Dissociative experiences scale (DES)

DES is a self-report questionnaire composed of 28 items that measures the likelihood of having dissociative thinking and post-traumatic stress disorder (PTSD) (Bernstein and Putnam, 1986). Measured on a 10-point scale (from “never” to “always”), each item describes a type of experience that each subject might have had. Studies show that subjects with scores above 15 need more exploration as they are often linked to dissociative diagnoses; a score of 30 suggests a good possibility of dissociative thinking and PTSD; finally, scores above 40 indicate high likelihood of a dissociative identity disorder (Kianpoor et al., 2012). Factor analysis reports three factorial structures (Burch, 1995) that include depersonalization and derealization (e.g., feeling that our body does not belong to us), amnestic dissociation (e.g., finding new objects that one does not remember buying), and imaginal involvement (e.g., being in a familiar environment but recognizing it as strange) (Renard et al., 2012). In reference to psychometric properties, Frischholz et al. (1992) report good concurrent and criterion validity, while Olsen and Beck (2012) show high internal consistency scores with Cronbach’s alpha of 0.7.

#### The Pittsburgh sleep quality index (PSQI)

PSQI is a self-administered questionnaire that measures sleep quality and quantity in 1 month (Buysse et al., 1989). The test consists of 19 items that address seven components; specifically: subjective quality and latency of sleep, duration, usual sleep efficiency, sleep disturbances, use of appropriate sleep medications, and schedules referred to sleep dysfunctionality. There are also five additional questions completed by the partner of the participant subject, but which are not then calculated in the final scoring. The Global Sleep Quality scale reports Cronbach’s alpha of 0.83. Furthermore, a Global Sleep Quality score greater than five discriminates between good and poor sleepers with a diagnostic sensitivity of 89.6 percent and specificity of 86.5 percent (Buysse et al., 1991). Finally, the literature evidences the reliability and validity of the PSQI in different populations (Beck et al., 2004).

#### Hospital and anxiety depression scale (HADS)

HADS is a 14-item compost questionnaire on a 4-point Likert scale (0–3) to measure emotional distress (e.g., anxiety and depression) in a patient population without psychiatric symptoms (Zigmond and Snaith, 1983). The total score (0–42) results from the sum of the scores obtained in each of the two individual subscales; the first subscale is about anxiety symptoms (7 items), while the second one regards depressive symptoms (7 items) (Zigmond and Snaith, 1983). The higher the score obtained, the higher the values found in reference to anxiety and depression; values with scores of 10 and above indicate the need for clinical intervention (Zilliacus et al., 2011; LoMartire et al., 2020). Studies designed to measure the applicability of this test to different populations (Bjelland et al., 2002; Cosco et al., 2012; Norton et al., 2013) show good reliability (Bjelland et al., 2002; Zigmond and Snaith, 1983) in both the general population and in samples of patients with chronic diseases (Lynch et al., 2011; Woolrich et al., 2006).

#### Functional assessment of cancer therapy—breast cancer (FACT-B), version 4

The FACT-B is a self-administered scale comprising 36 items designed to assess Quality of Life (27 items) (Cella et al., 1993; Di Bella et al., 2018) and specific aspects related to breast cancer, including body image and sexual satisfaction (9 items) (Bichoo et al., 2021). It evaluates different domains of Quality of Life, such as physical, emotional, and relational aspects. Participants rate their level of agreement on a 5-point Likert scale (ranging from “not at all” to “very much”) based on their experiences over the past 7 days. Higher scores indicate a better Quality of Life across all the domains explored (Deepa et al., 2020).

#### Objectified body consciousness scale (I-OBCS)

I-OBCS is a questionnaire consisting of 24 items from the original English version by McKinley and Hyde (1996). Participants are asked to indicate their degree of agreement on a 7-point Likert scale (from 1: very disagree to 7: very agree). I-OBCS measures surveillance attitudes toward the body, control beliefs about one’s physical appearance, and body shame (Boursier et al., 2020). Dakanalis et al. (2017) report good construct validity, high internal consistency, and test–retest reliability.

## Data analysis and sample size

The sample size required for the present study was calculated using G∗Power 3.1.9.2 software (Faul et al., 2007), for an analysis of variance with repeated measures (ANOVA) with interaction. The primary endpoint will be the difference between the two experimental groups (i.e., between-subjects factor) in the improvement of the overall MAIA scale score at pre-intervention (T0), post-intervention (T1), and follow-up (T2) times. To detect a weak-median effect size (i.e., partial η2 = 0.04), the required sample size is 40 (i.e., 20 participants in each experimental group). The type I error rate (*α*) was set at 0.05 (two-sided) and the power (1 - *β*) was set at 0.90. The collected data will be analyzed using the statistical analysis software Statistical Package for Social Science (SPSS, version 27.0).

Adherence to the intervention will be monitored and reported in the results, and data from participants who do not adhere will be managed using an intention-to-treat analysis.

The data collected in this study will be analyzed using repeated measures ANOVA to compare the outcomes between the experimental and control groups across the three time points. The primary analysis will focus on changes in the overall MAIA scale scores, allowing us to evaluate the main effects of time and group as well as the interaction between these factors. Secondary exploratory analyses will include repeated measures ANOVAs on the individual MAIA subscales and additional psychological outcomes such as anxiety, depression, and body image perception. Effect sizes and confidence intervals will be reported alongside *p*-values to offer a comprehensive understanding of the observed effects.

## Disadvantages or undesirable effects

No inconveniences or undesirable effects are expected for research participants. It will be the responsibility of the researchers to inform on the first presentation sheet of the study the possibilitỳ for participants to discontinue participation in the research at any time, without having to give any explanation and without incurring any possible negative consequences.

## Description of participants

The present study is aimed at women 18 years of age or older who have had breast cancer in the past. Participants will be recruited by email and will be informed about both the objectives and methods of the research. Regarding the questionnaires, a code will be assigned to render the collected data in a pseudonymous form. Firstly, each participant will be assigned a unique alphanumeric code, which will be used to identify them throughout the study. This code will replace any personally identifiable information in all study documents and databases, ensuring anonymity. Secondly, all data will be stored in a secure database protected by strong passwords, accessible only to authorized personnel directly involved in the study. Adherence to the study with informed consent will be requested prior to the administration of the questionnaires. Study participation is completely free, voluntary, and free of charge. All participants will have the option to discontinue the research at any time without the need to provide any explanation.
